# A synbiotic-containing amino-acid-based formula improves gut microbiota in non-IgE-mediated allergic infants

**DOI:** 10.1038/pr.2017.270

**Published:** 2017-12-06

**Authors:** David C A Candy, Marleen T J Van Ampting, Manon M Oude Nijhuis, Harm Wopereis, Assad M Butt, Diego G Peroni, Yvan Vandenplas, Adam T Fox, Neil Shah, Christina E West, Johan Garssen, Lucien F Harthoorn, Jan Knol, Louise J Michaelis

**Affiliations:** 1 Royal Alexandra Children’s Hospital, Brighton, UK; 2 Nutricia Research, Nutricia Advanced Medical Nutrition, Utrecht, The Netherlands; 3 Laboratory of Microbiology, Wageningen University, Wageningen, The Netherlands; 4 University Hospital Verona, Verona, Italy; 5 University Hospital Brussels, Brussels, Belgium; 6 Guy’s and St Thomas’ Hospitals NHS Foundation Trust, London, UK; 7 Great Ormond Street Hospital, London, UK; 8 Umeå University, Umeå, Sweden; 9 Utrecht Institute for Pharmaceutical Sciences, Faculty of Science, Utrecht University, Utrecht, The Netherlands; 10 Great North Children’s Hospital, Newcastle upon Tyne, UK

## Abstract

**Background:**

Prebiotics and probiotics (synbiotics) can modify gut microbiota and have potential in allergy management when combined with amino-acid-based formula (AAF) for infants with cow’s milk allergy (CMA).

**Methods:**

This multicenter, double-blind, randomized controlled trial investigated the effects of an AAF-including synbiotic blend on percentages of bifidobacteria and *Eubacterium rectale/Clostridium coccoides* group (ER/CC) in feces from infants with suspected non-IgE-mediated CMA. Feces from age-matched healthy breastfed infants were used as reference (healthy breastfed reference (HBR)) for primary outcomes. The CMA subjects were randomized and received test or control formula for 8 weeks. Test formula was a hypoallergenic, nutritionally complete AAF including a prebiotic blend of fructo-oligosaccharides and the probiotic strain *Bifidobacterium breve* M-16V. Control formula was AAF without synbiotics.

**Results:**

A total of 35 (test) and 36 (control) subjects were randomized; HBR included 51 infants. At week 8, the median percentage of bifidobacteria was higher in the test group than in the control group (35.4% vs. 9.7%, respectively; *P*<0.001), whereas ER/CC was lower (9.5% vs. 24.2%, respectively; *P*<0.001). HBR levels of bifidobacteria and ER/CC were 55% and 6.5%, respectively.

**Conclusion:**

AAF including specific synbiotics, which results in levels of bifidobacteria and ER/CC approximating levels in the HBR group, improves the fecal microbiota of infants with suspected non-IgE-mediated CMA.

Cow’s milk allergy (CMA) affects up to 5% of infants and children in Western countries (1, 2, 3), although the number of challenge-proven cases may be lower (4). CMA is associated with a range of distressing and potentially severe clinical symptoms affecting the skin, gastrointestinal (GI) tract, and, less commonly, the respiratory tract (5). Infants with non-IgE CMA generally have delayed symptoms (6) and present particular challenges because of difficulty in diagnosis, lack of validated tests, and a paucity of clinical studies (7). When exclusive breastfeeding is not possible, or there is failure of control of symptoms when dairy (±soya) is eliminated out of the maternal diet, the dietary management guidelines for infants with CMA recommend extensively hydrolyzed formula (eHF) for mild cases and amino-acid-based formula (AAF) for severe cases. When eHF is not tolerated and fails to resolve allergy symptoms, AAF is recommended (6, 8).

Aberrant composition and diversity of gut microbiota in early life may disrupt development of the immune system (9, 10, 11), which is associated with allergy-related diseases (12, 13, 14), including food allergies, such as CMA (15). A study of infants with CMA showed that gut microbiota composition at 3–6 months was associated with allergy resolution by the age of 8 years (16), suggesting that, during infancy, gut microbiota could be a potential mechanism to influence food allergy outcomes in childhood.

Studies showing that prebiotics and probiotics, or their combination (synbiotics), can positively modulate the composition of gut microbiota (17, 18, 19, 20, 21), provided the rationale to investigate such an approach in CMA. In addition, clinical studies of eHF supplemented with probiotics showed improved symptoms in infants with CMA (22, 23, 24, 25); however, eHF may not be the most appropriate formula for patients with non-IgE CMA, such as those presenting with faltering growth or those with persistent clinical symptoms when using eHF, (6) and an AAF is recommended in such cases (8). Clinical studies in healthy infants (26) and infants with CMA (26, 27) showed that synbiotic-supplemented AAF was hypoallergenic, well tolerated, and supported normal growth (26, 27). On the basis of these findings, we conducted a randomized trial to assess the effect of an AAF with a specific and optimized synbiotic blend on fecal microbiota composition and to explore clinical effectiveness in infants with suspected GI non-IgE-mediated CMA (28). This is the first randomized trial of a synbiotic-supplemented AAF exclusively in infants with suspected non-IgE CMA and includes an age-matched healthy breastfed reference group (HBR).

## Methods

### Trial Design

This was a multicenter, double-blind, randomized controlled trial (Netherlands Trial Register NTR3979) including subjects with suspected non-IgE CMA and a non-randomized reference group (HBR). Subjects were recruited by Great North Children’s Hospital, Newcastle; Barts/Royal Hospital, Guys and St Thomas’ Hospital, GOSH, London; Royal Alexandra Childrens Hospital, Brighton in the UK; University Hospital Padova, Padua; University Hospital Verona, Verona in Italy; CU St Luc, University Hospital Brussels, Huderf, Brussels in Belgium; and Umeå University, Umeå, Sweden. Eligible subjects, enrolled from 1 October 2013 to 30 April 2015, were stratified based on predominant, investigator-assessed symptoms (skin or GI) and randomly allocated to either test or control formula. The random allocation, by using a central Interactive Web Response System (Orca Pharma, Heesch, The Netherlands), was performed by a generated sequence/algorithm using block randomization to ensure that the test and control formulae were assigned equally. Formulae were identically packaged in 400 g tins and labeled with a one-letter code so that parents/guardians and those assessing outcomes were blind to the group assignment.

Test and control formulae were given for 8 weeks, after which the subjects were switched to a prescribed formula appropriate for their condition and age according to the local clinician’s choice and practice. Cow’s milk protein was introduced depending on local clinical practice. The subjects continued test or control formula if an AAF was considered the most appropriate approach for dietary management of clinical symptoms. Solids introduced in diets of the subjects were recorded by means of food diaries. The trial duration from screening to completion was a maximum of 28 weeks.

The trial was approved by the ethics committees of participating centers and all parents/guardians provided written informed consent.

### Participants

Subjects were randomized if they were aged <13 months and had a clinical history or strong suspicion of an allergic reaction to cow’s milk protein, based on a robust diagnostic work-up (Table 1) collectively designed by a multidisciplinary team of clinicians, comprising pediatric gastroenterology, allergy, and immunology specialists. The defined inclusion criteria (Table 1) included a negative specific IgE test (ImmunoCAP), and/or a negative skin-prick test with cow’s milk protein, if a test was performed (testing was not mandatory per protocol). In addition, at study entry, the subjects had at least one of the following (GI) symptoms related to inclusion of cow’s milk protein in their diet: faltering growth; frequent regurgitation or vomiting; extended periods of diarrhea with a negative stool examination (negative microbiology and virology laboratory tests); soft stool constipation; blood in stool; iron-deficiency anemia due to occult or macroscopic blood loss in stools not due to infection or dietary insufficiency; endoscopically confirmed eosinophilic enteropathy; or persistent distress or colic (>3 h per day at least 3 days per week over a 3-week period). Infants were excluded for the following reasons: birth weight <2,500 g, <37 weeks’ gestation requiring specific premature infant formula at study entry, severe concurrent illness, functional GI symptoms without suspicion of atopy and food allergy, immune, autoimmune, or gluten-sensitive enteropathy, food protein-induced enterocolitis syndrome, acute or chronic diarrhea secondary to a confirmed infectious gastroenteritis, behavioral disorders with food aversion or food phobia, GI surgery, syndromes commonly associated with functional GI disorders, and the use of probiotics, systemic antibiotics, or antimycotic drugs 4 weeks preceding study entry.

**Figure 1:**
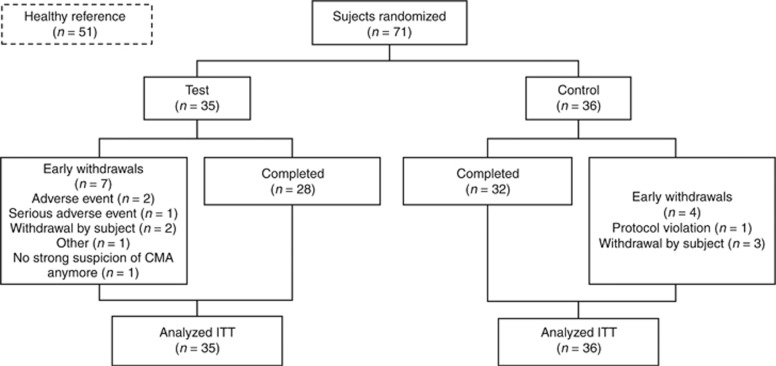
CONSORT diagram showing the flow of subjects in the randomized arms. ITT, intent to treat. Early withdrawal-related adverse events were constipation (*n*=1) and infantile colic (*n*=1), and related serious adverse event (*n*=1) was viral laryngitis. The events were reported as unlikely and not related to study formula.

Two weeks after randomization, symptom resolution was evaluated and subjects with persistent symptoms were reassessed by the investigator, and only subjects with suspicion of, or confirmed, non-IgE CMA continued in the study. Subjects not eligible at reassessment were withdrawn (Figure 1).

The non-randomized, HBR group comprised infants who were exclusively breastfed until 7 months of age. Healthy subjects of 7 months or older did consume solids, which were recorded in dietary diaries. In addition, the subjects did not have any concurrent illness or clinical history of allergy, did not receive any treatment or nutritional intervention, and were within a similar age range to subjects in the randomized groups. Infants in the HBR group were prospectively recruited from selected study centers and local community centers.

### Interventions in the Randomized Arms

The test formula was a hypoallergenic, nutritionally complete AAF (Neocate LCP; Nutricia Advanced Medical Nutrition, Liverpool, UK) containing a prebiotic blend of chicory-derived neutral oligofructose and long-chain inulin (BENEO-Orafti SA, Oreye, Belgium; 9:1 ratio at a total concentration of 0.63 g/100 ml) and a probiotic strain *Bifidobacterium breve* M-16 V (Morinaga Milk Industry, Tokyo, Japan) at a concentration of 1.47 × 10^9^ colony-forming units (CFU)/100 ml formula. The control formula was a commercially available AAF without synbiotics (Neocate LCP; Nutricia Advanced Medical Nutrition). Subjects were instructed to consume a minimum, age-specific, daily formula intake from the end of week 2 (infants aged 0–6 months, 500 ml; 6–8 months, 450 ml; and >9 months, 350 ml).

### Assessments

Medical history was documented by the clinician, for both test and control group, at baseline (week 0) and via 24-h recall of baseline presenting complaints. Stool samples for analysis were collected by parents/guardians at week 0, if possible under the supervision of a health-care professional, and then at home at weeks 8, 12, and 26. The samples collected into 10 ml stool containers (Greiner Bio-One, Kremsmünster, Austria) were immediately frozen at −80 °C in the clinic or at −20 °C in a home freezer before transferring it to the clinic storage facility. Parents/guardians completed a diary to record stool characteristics (frequency, color, and consistency; over 3 days during weeks 1, 4, 8, 12, and 26), study formula intake (volume consumed over 7 days during weeks 1, 4, and 8), and diet evaluation (type of foods eaten by the subject at the end of each week during weeks 1, 4, 8, 12, and 26). Skin symptoms (including atopic dermatitis), respiratory symptoms (blocked nose, coughing, and wheezing), GI symptoms (vomiting, spitting up), and general symptoms (ease to settle or burp after feeds, and visual signs of discomfort, e.g., back arching and crying due to irritability) were recorded in the diary (collected over 3 days during weeks 1, 4, 8, 12, and 26) and reviewed by the investigator during clinic visits. In order to collect HBR stool samples that could be age-matched with the week 8 age range of the CMA infants, stool samples from HBR were collected at one time point, or more if feasible for infant and parents. The stool sample collection was as described above and accompanied with a completed diet diary and stool characteristic assessment. After study completion, and before deblinding the groups, age-matching HBR samples were selected for reference analyses.

### Objectives and Outcomes

The primary objective was to assess the effect of test formula on fecal percentages of bifidobacteria and *Eubacterium rectale/Clostridium coccoides* group (ER/CC) at 8 weeks, determined by florescence *in situ* hybridization analysis using 16S rRNA-targeted oligonucleotide probes, as described previously (29). Bifidobacteria are typically abundant in healthy breastfed infants (30) and show stable and increased levels in early childhood compared with adults (31). As maturation to adult-like profiles may extend beyond 5 years of age, ER/CC was selected as a marker because it typically is one of the first adult-like bacterial groups appearing in the infant gut (30, 31).

Assessing the effects of test formula on stool characteristics at weeks 0 and 8 was a secondary objective.

Measuring clinical effectiveness of test formula on allergic symptoms was an exploratory objective. Skin symptoms at weeks 0 and 8 were evaluated by the SCORing Atopic Dermatitis (SCORAD) rating scale (32). Parent-reported rating scales for skin, respiratory, GI, and general symptoms were collected using a four-point scale, where a score of 1 was taken as normal with no symptoms. Assessed parent-reported symptoms were evaluated together with the clinician during visits.

The frequency and severity of adverse events and use of concomitant medication were used to assess safety and tolerability. Standard anthropometric measurements were recorded to assess growth.

### Statistics

Sample size estimation was based on effect size difference of 26.4% in bifidobacteria and 23.6% in the ER/CC group (29). Assuming a significance level of 5% using a two-sided statistical test and Hochberg principle for two parameters, a sample size of 20 completers per study arm gave 80% power to observe an effect. Assuming estimated drop-out rates of up to 25% of subjects whose symptoms did not resolve within 2 weeks of starting AAF and 20% for other reasons, 68 subjects were to be recruited. Following a semi-blinded interim analysis by an independent committee, it was decided to keep the sample size unchanged. This committee consisted of a clinical study expert, a gut microbiology expert, and a statistician. These experts were not involved in any discussion or decision regarding conduct of the study or study results after they evaluated semi-blinded data.

Overall statistical analyses were performed comparing test with the control group. To bring microbiota outcomes in a context of a healthy situation, levels of a reference group (HBR) were determined and used as reference only and not as a treatment group. Two primary outcome parameters (bifidobacteria and ER/CC) were recorded as percentages of total fecal bacteria. All analyses were performed on intention-to-treat data set (ITT), defined as all randomized subjects. Analysis of covariance (ANCOVA) was performed with a between-subjects factor “group” (test vs. control) and both the stratification factor (skin or GI) and the baseline measure as covariate (the primary model). The model-based intervention effect size was calculated, and the significance of the fixed parameter “group” estimate was evaluated. To evaluate potential influence on primary outcome, additional sensitivity analyses were performed for the predetermined covariates: age at baseline, mode of delivery, sex, antibiotic use during study, introduction of weaning foods, total duration of breastfeeding, intake of proton pump inhibitors or H2 antagonists, study site, and country. For sensitivity analysis 1 (including only age at baseline as covariate) the estimate was compared with the estimate of the primary model; for sensitivity analysis 2 (including age at baseline and one of the other covariates) the estimate was compared with the estimate of sensitivity analysis 1.

Secondary and exploratory outcome parameters were reported descriptively. *P* values for SCORAD (change from baseline), clinical symptoms, and stool frequency (levels at week 8) were based on the Van Elteren test comparing test and control groups accounting for the stratification factor (skin or GI symptoms). *P* values for the mean daily formula intake were tested by *t*-test and the median daily intake by means of Wilcoxon sum-rank test.

Statistical analyses were performed by using SAS (SAS Enterprise Guide version 4.3 or higher) for Windows (SAS Institute, Cary, NC). Results are expressed as mean values±SD unless stated otherwise.

## Results

A total of 71 subjects with suspected non-IgE CMA were recruited from 11 centers in the UK, Italy, Belgium, and Sweden. Figure 1 summarizes the flow of patients in the randomized treatment arms; 35 subjects were randomized to test formula and 36 to control formula. Early withdrawal-related adverse events were constipation (*n*=1) and infantile colic (*n*=1), and a related serious adverse event (*n*=1) was viral laryngitis. The events were reported as unlikely and not related to study formula.

A total of 110 stool samples from 60 healthy subjects were collected, and 51 subjects were considered eligible following predefined criteria; subsequently, 51 stool samples from these subjects were selected as healthy reference samples by age-matching with age of CMA subjects at week 8 of intervention (Table 2). Characteristics of subjects in the randomized treatment arms were well balanced with respect to baseline demographics—except for the mode of delivery, which was twice as high in the control group compared with that in the test group (Caesarean section 41.7% and 20.0%, respectively; Table 2). Most subjects were Caucasian and 60% were recruited in the UK, whereas Sweden contributed most infants in the HBR group. Most CMA subjects suffered from the following symptoms associated with cow’s milk protein ingestion: frequent regurgitation or vomiting (72%), persistent distress or colic (70%), eczema (52%), a change in behavior such as irritability or crying (44%), soft stool constipation (41%), and faltering growth (34% Table 3). GI, skin, respiratory, and other symptoms were well balanced at baseline. GI symptoms were the predominant complaint in 90.1% of the subjects, whereas the remainder suffered predominantly from skin symptoms. Most infants in test (29%, 46%) and control groups (36%, 53%) were already on hydrolysate formula or AAF, respectively, at study entry. The average amount of study formula intake during the study did not differ between study groups. In week 1, the mean daily intake (±SD) was 602 (±247) and 627 (±205) ml in test and control group, respectively (*P*=0.646). The mean daily intake for test and control groups were 629 (±213) and 660 (±238) ml (*P*=0.603) and 652 (±176) and 639 (±212) ml (*P*=0.797) in weeks 4 and 8, respectively. Nature and frequency of solid foods consumed during the study were well balanced between test and control groups as well as between the HBR group and both CMA groups at week 8 (data not show).

**Figure 2:**
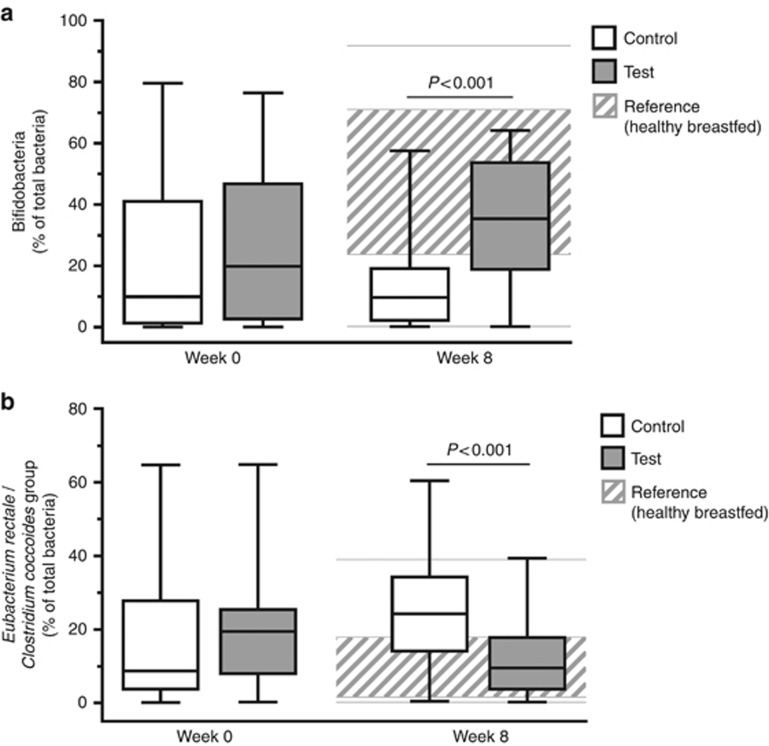
Percentages of bifidobacteria (**a**) and adult-like *ER/CC* (**b**) at weeks 0 and 8 in subjects given test formula or control formula (ITT). The gray shaded area represents the sample 25th to 75th percentile of the healthy reference group (healthy, breastfed subjects), and the gray horizontal lines represent the minimum and maximum values of the healthy reference group. *P* values are based on ANCOVA comparing test vs. control groups with week 8 values as outcome, stratification factor (skin or gastrointestinal symptoms) and imputed baseline values as covariate and intervention as fixed effect, respectively.

The primary outcome showed statistically significant differences (*P*<0.001) between test and control groups at week 8 in the fecal composition of bifidobacteria and ER/CC (Figure 2). In subjects given AAF including synbiotics, the median percentages of bifidobacteria were higher at week 8 compared with those in the control group (35.4% vs. 9.7%, respectively), whereas the median percentages of adult-like ER/CC were lower (9.5% vs. 24.2%, respectively). The differences between test and control arms were statistically significant for bifidobacteria (+20.937% (95% confidence intervals 10.14, 31.74); *P*<0.001) and ER/CC (−14.115% (−22.21, −6.02); *P*<0.001). At week 8, the median percentages of bifidobacteria and ER/CC of the test group were 35.4% and 9.5%, respectively, which approximated the levels in the HBR (55% and 6.5%) more so than the levels in the control group (9.7% and 24.2%). The sensitivity analyses considered intake of proton pump inhibitors or H2 antagonists as potential confounder for bifidobacteria analyses and antibiotic use and sex as potential intervention effect modifiers. Including intake of proton pump inhibitors or H2 antagonists as an additional covariate into the ANCOVA model, however, did not change the outcome of the primary parameters bifidobacteria (*P*<0.001) and ER/CC (*P*<0.001) at week 8. A subgroup analysis on subjects who did not take any systemic antibiotics (*n*=47) showed that differences between test and control groups were statistically significant for both bifidobacteria (*P*<0.001) and ER/CC (*P*<0.001; Supplementary Figure S1 online). In addition, the differences between the test and control groups at week 8 were also statistically significant in both males (*n*=41) and females (*n*=15) for bifidobacteria (*P*=0.037 and *P*<0.001, respectively) and ER/CC (*P*=0.047 and *P*=0.032, respectively; Supplementary Figure S1). The sensitivity analyses showed that all other predetermined covariables—including age at baseline, mode of delivery, introduction of weaning foods, total duration of breastfeeding, study site, and country—did not significantly influence primary outcome.

Stool frequency score was lower in the test group than in the control group (1.88±0.19 vs. 1.98±0.15; *P*=0.015); however, all other measures of stool characteristics were not statistically significantly different between groups at week 8 (data not shown).

**Figure 3:**
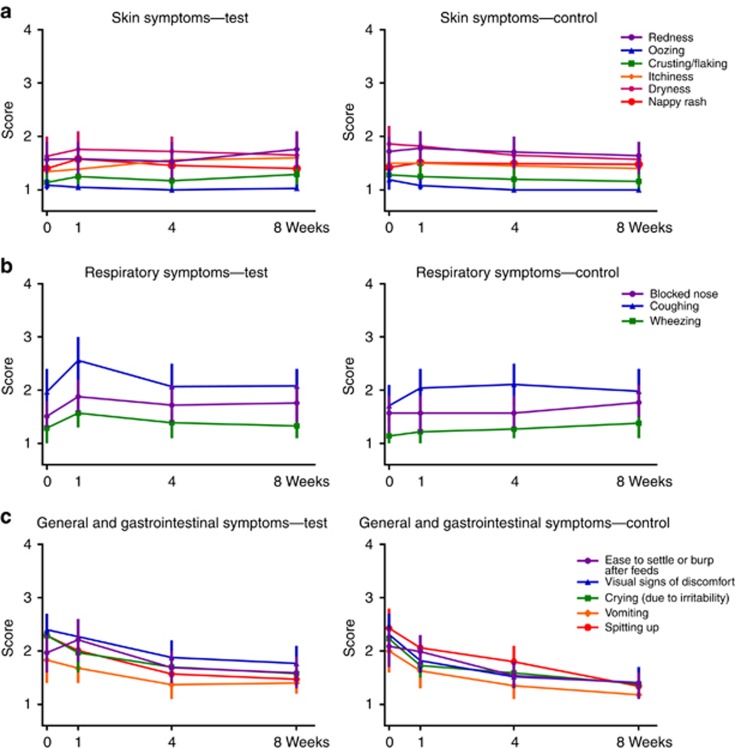
Parent-reported, clinician-evaluated symptoms at weeks 0, 1, 4, and 8 assessed on a 4-point rating scale specific for each symptom. Skin symptoms (redness, oozing, crusting, itchiness, dryness, and nappy rash) were rated as 1: none, 2: slight, 3: some, and 4: a lot. Respiratory symptoms blocked nose and wheezing rated as 1: none, 2: mild, 3: moderate, and 4: severe, and coughing was rated as 1: none, 2: one to two times/day, 3: three to five times/day, and 4: more than five times/day. General and gastrointestinal symptoms: vomiting was rated as 1: none, 2: one to two times/day, 3: three to four days/day, and 4: more than four times/day; spitting-up as 1: none, 2: after some feeds, 3: after all feeds, and 4: between and after feeds; gas/wind as 1: none; 2: slight; 3: some; and 4: a lot; sleep pattern last night as 1: normal, 2: awake once, 3: awake two to three times, and 4: awake more than three times; ease of settling or burping after feeds as 1: no problem at all, 2: slight difficulty, 3: some difficulty, and 4: very difficult; visual signs of discomfort (e.g., back arching) as 1: none, 2: slight, 3: some, and 4: a lot; and crying (because of irritability) as 1: none, 2: up to 1 h, 3: 1–3 h, and 4: more than 3 h. Data are shown as mean values±95% confidence interval limits.

In exploratory analyses of clinical outcomes, no statistically significant differences were observed at week 8. Figure 3 shows clinical symptoms reported at weeks 0, 1, 4, and 8. GI symptoms were predominant in the study population. Reported scores for GI and general symptoms were, compared with other assessed outcomes, relatively high at baseline (2.0–2.5) and decreased over time (Figure 3). In contrast, reported scores for skin symptoms were relatively mild at baseline (generally below 1.5), and showed no statistically significant changes over 8 weeks. SCORAD decreased between weeks 0 and 8, from 12.83±18.84 to 9.63±12.45 in the test group and from 14.43±19.74 to 7.06±10.01 in the control group.

Overall, there were no differences in the number of subjects reporting adverse events between test and control groups during the first 8 weeks (Table 4). The total number of concomitant medications taken was lower in subjects given test formula (82) compared with those given control (111) formula, although the number of subjects (N=21 vs N=28, respectively) was not statistically significant between arms. Further evaluation showed a significantly lower percentage of subjects in the test group needed medication related to the subcategory designated “systemic anti-infectives” (subgroup according to the Anatomical Therapeutic Chemical coding system, which includes antibacterials for systemic use and vaccines) compared with that in the control group (8.6% vs. 34.4%, respectively; *P*=0.018).

Growth parameters were within the expected ranges for age and median *Z*-scores for both groups were within 1 SD of the mean (data not shown).

## Discussion

The primary objective of modifying gut microbiota using an AAF including a blend of prebiotic fructo-oligosaccharides and the probiotic strain *B. breve* M-16V for 8 weeks in subjects with suspected non-IgE CMA was achieved. Percentages of bifidobacteria were higher and adult-like ER/CC lower among infants given the AAF with these specific synbiotics compared with those given the AAF without synbiotics.

This study was primarily designed to investigate whether the synbiotic ingredients can improve the gut microbiota in infants with non-IgE CMA to achieve a microbial composition close to that seen in healthy, breastfed infants. Previous studies showing the effects of breast milk on gut microbiota (33, 34) helped to guide the development of this AAF, which contains a specific blend of prebiotics and probiotics. *B. breve* is a bacterial species found in human milk and the gut of healthy infants (33, 35), and in preclinical models *B. breve* M-16V was identified as the most potent anti-allergic probiotic strain tested (36). Another model of established food allergy showed potential immunomodulatory benefits of dietary intervention with a synbiotic combination of olifructose and inuline (short- and long-chain fructo-oligosaccharides) and *B. breve* M-16V (21). The current study showed that microbial composition of infants with suspected non-IgE CMA who received the test formula was closer to the profile of the HBR group than those infants receiving control formula. On the basis of previous studies, we hypothesized that modifying the gut microbiota with these specific synbiotics may improve clinical symptoms associated with gut microbiota dysbiosis and dysregulated immune reactions in infants with CMA (37, 38). Overall, exploratory GI and general symptoms improved over time, but were not statistically significantly different between test and control groups at week 8. This trial was not primarily designed or powered to show differences in clinical outcomes between groups, and it is important to note that the majority of subjects were already receiving a hydrolyzed formula or AAF at study entry, which confounds interpretation of the exploratory data analysis.

This randomized trial has several limitations. The trial was designed to evaluate the effects of AAF with synbiotics exclusively in subjects with non-IgE CMA. There is no standard test for the precise diagnosis of non-IgE allergy, raising the possibility that subjects with other conditions, for example, other (food) allergy presentations, could dilute the trial population. The possibility of erroneous entry into the trial was addressed by using a robust diagnostic work-up (Table 1) collectively designed by a multidisciplinary team of clinicians, comprising pediatric gastroenterology, allergy, and immunology, and was based on careful symptom assessment by the investigators, with specific IgE testing and skin-prick testing (if assessed) to exclude any IgE-mediated CMA. Additional research is warranted to define precise biomarkers for this allergic phenotype.

Consistent with scientific methodology, it is essential to ensure that patients meet diagnostic criteria for eligibility and undergo a double-blind placebo-controlled challenge to confirm symptomatology to cow’s milk protein. Furthermore, as seen in clinical practice, determining disease resolution requires re-introduction of the food-by-food challenge or introduction at home. In the present trial, it was not mandatory for study subjects to have a food challenge to confirm CMA diagnosis, although this would have been ideal for the interpretation of the clinical outcomes. However, overall, in the context of our understanding of the many influences on the gut microbiota, the investigators do not believe that this specific limitation of the study would have a meaningful influence on the primary outcome.

One of the factors associated with microbiota development is the mode of delivery (i.e., caesarean or vaginal delivery) of an infant. In our study, twice as much caesarean-delivered infants were randomized to the control group compared with test group (42% and 20%, respectively) and could, therefore, potentially influence the primary outcome. However, our statistical analyses showed that this factor did not influence current study outcome.

Furthermore, there is no recognized standard profile for the composition of a healthy microbiota during the dietary diversification period in early life. The trial design partly addresses this issue by using an HBR group to allow age-matched comparison of data; however, it is important to recognize that the reference population is not identical to the randomized groups, for example, with respect to predominant country of origin, mode of delivery (14% caesarean section-born vs. 20% and 42% in test and control group, respectively), general health status, allergic symptoms, and dietary management. The authors feel that despite these limitations, this HBR group is a good representation of a healthy microbiota to function as reference. The duration of formula administration and length of follow-up mean there is only limited scope for this trial to assess longer-term changes in gut microbiota.

This randomized controlled trial adds to the evidence base for prebiotics and probiotics in the alteration of infant microbiota and potentially the dietary management of CMA (22, 23, 24, 25, 27, 39, 40, 41, 42). However, caution is required in making comparisons between studies, which had different trial entry criteria and tested a range of dietary management strategies. The test formula in this trial was a hypoallergenic, nutritionally complete AAF with a specific composition of prebiotics and probiotics, not derived from cow’s milk ingredients. The formula composition was similar to the one tested by Harvey *et al.* (26), but excluded the pectin-derived acidic oligosaccharides. Data with this formula cannot be extrapolated to other types of formula, e.g., eHF, or formulae containing different types of prebiotics or alternative strains of probiotics.

Safety concerns have been expressed with other infant formulae containing different probiotics (43, 44), and it is important that safety is established in clinical trials for each specific formula in an appropriate population (45). The synbiotic-supplemented AAF in this trial was shown to be safe in terms of adverse events, use of concomitant medications, and achievement of growth targets (26). Several studies have shown that *B. breve* M-16V is safe in infants, including preterm neonates (26, 27, 46). Although the addition of these specific synbiotics to AAF improves the composition of the gut microbiota so that it more closely resembles the composition observed in breastfed individuals, the results of this trial do not change the recommendation that infants with CMA should be fed with human breast milk if possible.

Although these results are specific to subjects with non-IgE-mediated CMA, Burks *et al.* (28) showed that an AAF, including ingredients from the current synbiotic blend, was safe in patients with IgE and non-IgE-mediated CMA. An ongoing clinical study (Netherlands Trial Register NTR3725) includes infants with confirmed IgE-mediated CMA, randomly allocated to receive AAF with or without synbiotics for 12 months, and will assess cow’s milk tolerance acquisition over 24 months. The ongoing and reported trial will inform future studies primarily focusing at clinical outcomes in the specific CMA populations.

On the basis of the data showing significant effects on the composition of gut microbiota that extend beyond an increase in bifidobacteria, we conclude that the AAF including the specific synbiotics of fructo-oligosaccharides and *B. breve* M-16V used in this trial was equally well tolerated as AAF without synbiotics, suitable for dietary management, and supports microbiota development of infants with suspected non-IgE-mediated CMA.

## Figures and Tables

**Table 1:** Inclusion and exclusion criteria

Inclusion criteria	Exclusion criteria
–Infants <13 months of age	–Infants <2,500 g at birth
–Clinical history or strong suspicion of an allergic reaction to cow’s milk protein with at least one of the following gastrointestinal symptoms:	–Infants <37 weeks of gestation requiring premature formula at study entry
(i) Chronic poor weight gain after dietary inclusion of cow’s milk protein	–Infants with severe concurrent illness
(ii) Frequent regurgitation or vomiting, whereby symptoms are related to the cow’s milk protein	–Infants with functional gastrointestinal symptoms, where atopy and food allergy is not suspected
(iii) Extended periods of diarrhea with a negative stool examination (lab test-negative)	–Infants with (auto)immune and gluten-sensitive enteropathy
(iv) Soft stool constipation^a^ (with/without perianal rash not due to infection)	–Infants with FPIES^b^
(v) Blood in stool	–Behavioral disorders with food aversion or food phobia
(vi) Iron deficiency anemia due to occult or macroscopic blood loss in stool not due to infection	–Infants who have acute chronic diarrhea secondary to confirmed infectious gastroenteritis (lab test-positive)
(vii) Endoscopically confirmed eosinophilic enteropathy	–Infants who have undergone gastrointestinal surgery (e.g., bowel resection, stoma)
(viii) Persistent distress or colic (>3 h/day, at least 3 days/week over a 3-week period)	–Infants with Down’s syndrome or other syndromes, where functional gastrointestinal disorders are common
–If performed results of specific IgE tests and/or SPT for cow’s milk protein are negative	–Use of probiotic bacteria or probiotic containing drinks/supplements/formula 4 weeks before study
–Expected minimum study formula intake per day at the end of week 2, 500 ml (0–6 months), 450 ml (6–8 months), and 350 ml (≥9 months)	–Use of systemic antibiotics or antimycotics 4 weeks before study

FPIES, Food Protein-Induced Enterocolitis Syndrome.

a Soft stool constipation is a term used when a subject uses excessive straining to pass liquid or soft stool (with an occasional hard plug).

b FPIES, which is associated with very severe symptoms, was excluded to reduce subject heterogeneity.

**Table 2:** Demographics of subjects with CMA and the healthy reference group

	Test (*N*=35)	Control (*N*=36)	Total CMA (*N*=71)	Healthy subjects (*N*=51)
*Age at baseline (months)*				
Mean (SD)	5.67 (3.24)	6.33 (2.71)	6.00 (2.98)	7.84 (3.25)
Min—Max	1.8–12.8	1.2–11.6	1.2–12.8	2.6–14.2
				
*Sex (%)*				
Female	28.6	25.0	26.8	45.1
Male	71.4	75.0	73.2	54.9
				
*Race (%)*				
Asian	5.7	2.8	4.2	0.0
Black	2.9	0.0	1.4	0.0
Caucasian/White	88.6	88.9	88.7	92.2
Combination of above/other	2.9	8.3	5.6	7.8
				
*Mode of delivery (%)*				
Caesarean section	20.0	41.7	31.0	13.7
Vaginal	80.0	58.3	69.0	86.3
				
*Country of residence (%)*				
Belgium	17.1	13.9	15.5	0.0
United Kingdom	60.0	69.4	64.8	29.4
Italy	17.1	13.9	15.5	11.8
Sweden	5.7	2.8	4.2	58.8

CMA, cow’s milk allergy. Healthy subjects: healthy breastfed reference group.

N is number of subjects. Denominator for % is number of subjects in the treatment group with non-missing data.

**Table 3:** Medical history of presenting complaints of subjects in the randomized arms at study baseline

Medical history of presenting complaints as examined by clinician, *N* (%)	Test (*N*=35)	Control (*N*=36)	Total (*N*=71)
*Overall symptoms*			
A change in behavior such as irritability or crying	12 (34.3%)	19 (52.8%)	31 (43.7%)
			
*Gastrointestinal symptoms*			
Frequent regurgitation or vomiting related to cow’s milk protein	23 (65.7%)	29 (80.6%)	52 (72.3%)
Persistent distress or colic (>3 h/day ≥3 days/week over a 3-week period)	24 (68.6%)	25 (69.4%)	49 (69.0%)
Soft stool constipation (with/without perianal rash due to infection)	12 (34.3%)	17 (47.2%)	29 (40.8%)
Faltering growth after the dietary inclusion of cow’s milk protein	13 (37.1%)	11 (30.6%)	24 (33.8%)
Extended periods of diarrhea with a negative stool examination	8 (22.9%)	9 (25.0%)	17 (23.9%)
Blood in stool	10 (28.6%)	5 (13.9%)	15 (21.1%)
Endoscopically confirmed eosinophilic enteropathy	0	0	0
			
*Skin symptoms*			
Eczema	16 (45.7%)	21 (58.3%)	37 (52.1%)
Urticaria	3 (8.6%)	4 (11.1%)	7 (9.9%)
			
*Respiratory symptoms*			
Sneezing/nasal congestion	9 (25.7%)	12 (33.3%)	21 (29.6%)
Wheezing	5 (14.3%)	5 (13.9%)	10 (14.1%)
Conjunctivitis	1 (2.9%)	3 (8.3%)	4 (5.6%)
Dyspnea	1 (2.9%)	1 (2.8%)	2 (2.8%)
Stridor	1 (2.9%)	1 (2.8%)	2 (2.8%)
Dysphonia	0	0	0
Aphonia	0	0	0
			
*Other symptoms*			
Hypotension for age	0	0	0
			
*Predominant complaint/stratification factor*			
Skin symptoms	4 (11.4%)	3 (8.3%)	7 (9.9%)
Gastrointestinal symptoms	31 (88.6%)	33 (91.7%)	64 (90.1%)

**Table 4:** Adverse events and use of concomitant medications in test and control groups

	Test (*N*=35)	Control (*N*=36)	*P* value (Fisher’s exact test)
*Adverse events, N (%)*			
Overall			
Any adverse event	20 (57.1%)	23 (65.7%)	0.624
Severity			
Mild	15 (42.9%)	15 (42.9%)	
Moderate	4 (11.4%)	7 (20.0%)	
Severe^a^	1 (2.9%)	1 (2.9%)	
Preferred term description^b^			
Gastrointestinal disorders	11 (31.4%)	13 (37.1%)	0.802
Infections and infestations	10 (28.6%)	12 (34.3%)	0.797
*Concomitant medication, N (%)*			
Overall			
Any concomitant medication	21 (60.0%)	28 (80.0%)	0.117
Subcategory^c^			
Anti-infectives for systemic use	3 (8.6%)	12 (34.4%)	0.018

a Reported severe adverse events were feeding disorder of infancy or early childhood (test group) and bronchiolitis and feeding disorder of infancy or early childhood (control group).

b The two most frequently reported preferred terms of adverse event are shown.

c Only categories (of total nine categories) with a statistically significant difference (*P*<0.05) are shown.
