# Supplementary figures and images for: A synbiotic-containing amino-acid-based formula improves gut microbiota in non-IgE-mediated allergic infants
Source: Pediatr Res. 2017 Dec 6;83(3):677–86. doi: 10.1038/pr.2017.270 (PMC6023699; doi:10.1038/pr.2017.270)

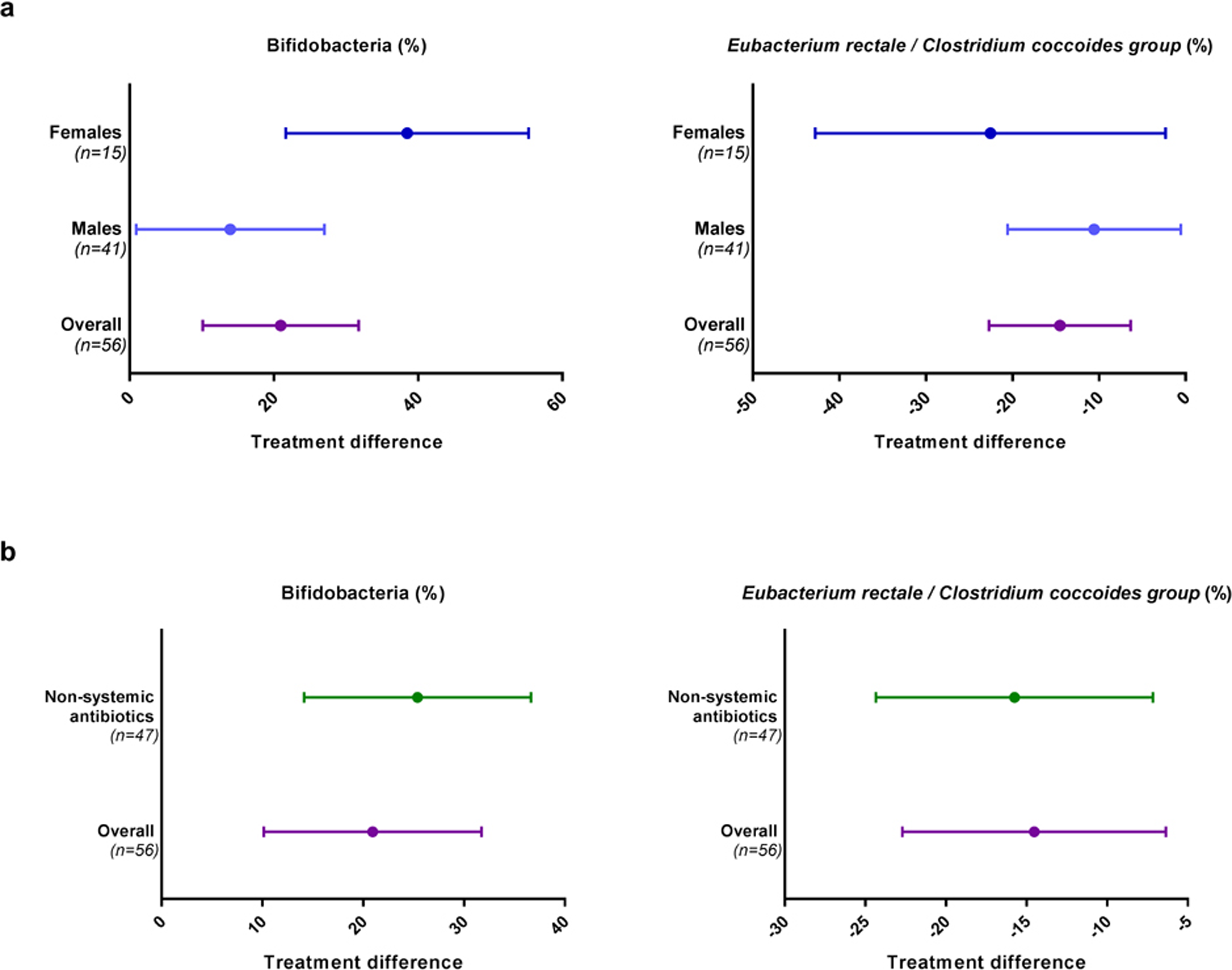

Supplement: Supplementary Figure S1 [file pr2017270x1.tif]
